# Comparison of attribute-based encryption schemes in securing healthcare systems

**DOI:** 10.1038/s41598-024-57692-w

**Published:** 2024-03-26

**Authors:** Redwan Walid, Karuna Pande Joshi, Seung Geol Choi

**Affiliations:** 1 Department of Information Systems, University of Maryland, Baltimore County, MD 21250 USA; 2 Department of Computer Science, United States Naval Academy, Annapolis, MD 21402 USA

**Keywords:** Attribute-based encryption, Attribute-based access control, Electronic health record, Knowledge graph (ontology), Health policy, Health services, Computer science, Information technology, Software

## Abstract

E-health has become a top priority for healthcare organizations focused on advancing healthcare services. Thus, medical organizations have been widely adopting cloud services, resulting in the effective storage of sensitive data. To prevent privacy and security issues associated with the data, attribute-based encryption (ABE) has been a popular choice for encrypting private data. Likewise, the attribute-based access control (ABAC) technique has been widely adopted for controlling data access. Researchers have proposed electronic health record (EHR) systems using ABE techniques like ciphertext policy attribute-based encryption (CP-ABE), key policy attribute-based encryption (KP-ABE), and multi authority attribute-based encryption (MA-ABE). However, there is a lack of rigorous comparison among the various ABE schemes used in healthcare systems. To better understand the usability of ABE techniques in medical systems, we performed a comprehensive review and evaluation of the three popular ABE techniques by developing EHR systems using knowledge graphs with the same data but different encryption mechanisms. We have used the MIMIC-III dataset with varying record sizes for this study. This paper can help healthcare organizations or researchers using ABE in their systems to comprehend the correct usage scenario and the prospect of ABE deployment in the most recent technological evolution.

## Introduction

### Cloud-based electronic health record systems and privacy regulations

Cloud infrastructure permits efficient and economical deployment of Big Data applications that can be accessed from any device platform. Cloud facilitates easy collaboration among team members as they can access the same infrastructure and work simultaneously even when geographically separated. Thus, medical organizations are increasingly adopting Cloud architecture to host their patient-facing applications, like the electronic health record (EHR) systems. As medical data is personal and sensitive, this transition must ensure data privacy and security of the EHR; else, patients may suffer adverse consequences from medical data breaches, such as job loss, health insurance, psychological harm, emotional distress, etc. The Health Information Technology for Economic and Clinical Health (HITECH) Act^1^ establishes privacy requirements that every healthcare provider must meet to deliver medical services. Moreover, the Health Insurance Portability and Accountability Act of 1996 (HIPAA)^2,3^ controls the administration and distribution of medical information by setting rules for maintaining the security and privacy of medical health data. Cloud-based EHR systems must abide by these legal requirements. They must guarantee data security and a smooth user experience. They should also put strong access control measures to avoid illegal access to the EHR systems.

### Attribute-based encryption and cloud-based EHR systems

Attribute-based encryption (ABE) has been a popular choice to address privacy risks linked with healthcare data. ABE offers vital features such as fine-grained access control and integrity preservation that are essential for addressing privacy and security. ABE has also proven structural efficiency, including quicker key generation, reduced computation time, fewer key pairs, and collision resistance^4^.

There are several types of ABE techniques, and the most popular among them are ciphertext policy attribute-based encryption (CP-ABE)^5^, key policy attribute-based encryption (KP-ABE)^6^, and multi authority attribute-based encryption (MA-ABE)^7^. CP-ABE embeds access policies directly into ciphertexts, allowing medical users to decrypt based on their possession of attributes that satisfy the policy. In KP-ABE, access policies are associated with secret keys and medical users are granted decryption capabilities if their key attributes match the attributes specified in the ciphertext. MA-ABE introduces multiple authorities responsible for attribute assignment, accommodating scenarios where diverse entities control different attribute sets. Most cloud-based attribute-controlled EHR systems in the current literature^8–14^ use either of the three underlying ABE schemes for addressing privacy and security in their system and enabling fine-grained access control.

A significant challenge in ”non-ABE” approaches involves addressing scalability issues when users have the flexibility to define their privacy settings. Consider a scenario where an individual intends to share their electronic health record (EHR) with various subgroups, such as friends or relatives. In utilizing different group keys, they are required to encrypt multiple copies of the data and manage the credentials of the groups granted access. This approach proves inadequate for achieving scalability. Despite the existence of several conventional public key encryption methods with granular access control, the necessity to encrypt numerous copies for diverse entities persists, leading to substantial key management costs^15^. Consequently, Attribute-based encryption (ABE) emerges as an optimal solution in such scenarios.

**Figure 1:**
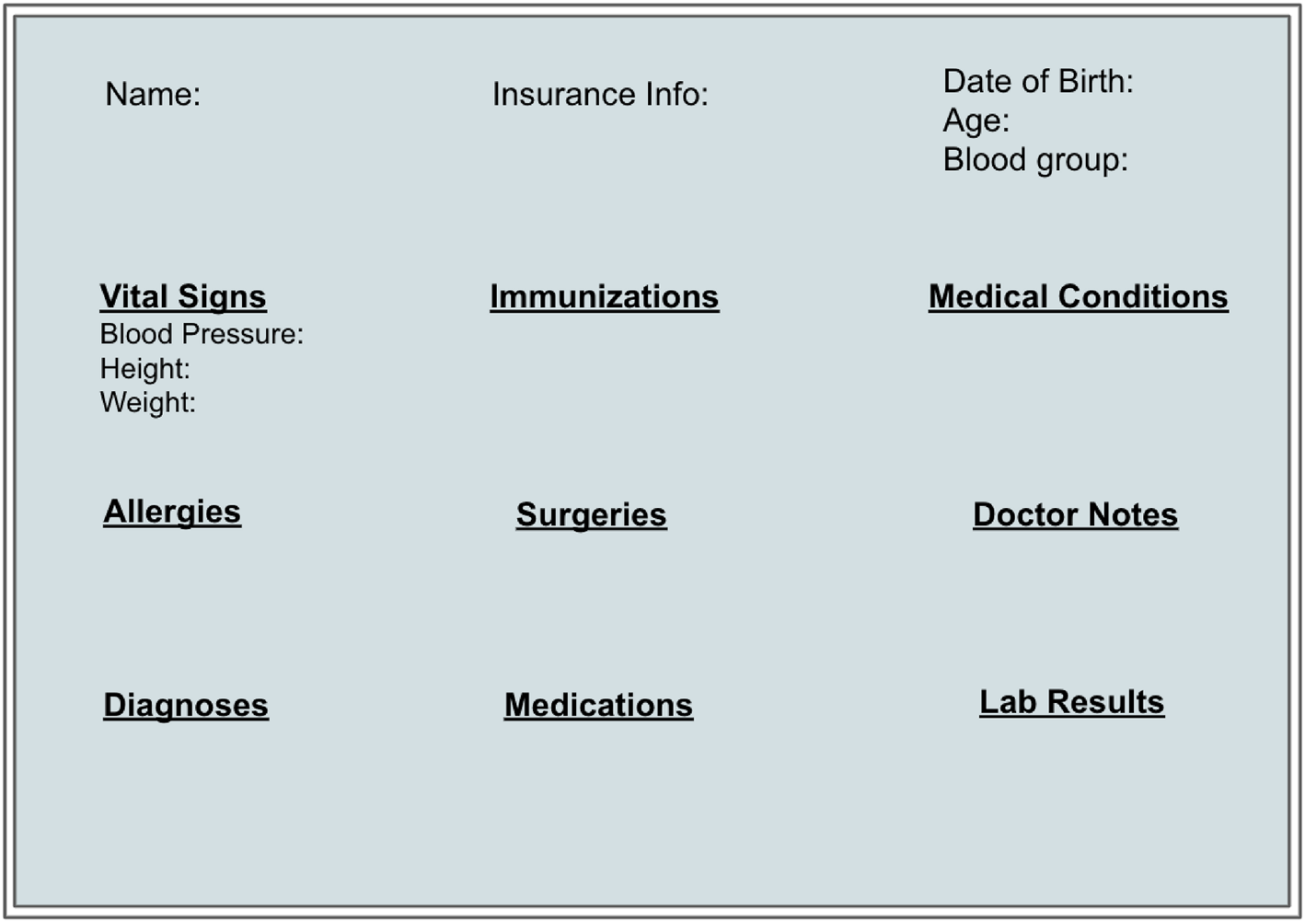
User interface of the EHR systems used for storing patient data.

### Our contributions

While attribute-based encryption (ABE) is widely adopted in electronic health record (EHR) systems, the specific ABE techniques employed vary across different systems. This paper conducts a detailed analysis of the three most popular ABE schemes utilized in contemporary EHR systems. Our contributions are as follows:

- We developed frameworks utilizing knowledge graphs and the MIMIC-III dataset, each implemented with a distinct ABE scheme. In particular,
  - We first identified the required information fields in a typical EHR system based on the HL7 EHR Functional Model^16^. The fields shown in Fig. 1 were used as references in our systems.
  - Since our systems design approach involves integrating semantic web technologies with ABE schemes, we first created knowledge graphs for each system that represent the entities of a medical organization. The knowledge graphs show the numerous EHR fields of patients, their associated properties, and the connections between various organizational units.
  - We created an attribute-based access control (ABAC) system^17^ that uses user attributes stored in the knowledge graphs to determine access permission. The HIPAA medical information storage and management policy serves as the foundation for our access policy regulations. For each system, we implemented an ABE encryption scheme on the data and stored them as encrypted nodes in the knowledge graph.
- We evaluated the performance of various queries in each system and examined the number and size of the public and private keys associated with each ABE scheme. These comparisons offer insights into which solution is most suitable for specific scenarios based on their usability within healthcare systems.
- We additionally provide a comprehensive examination of ongoing research efforts aimed at enhancing data security and privacy in EHR systems through the implementation of ABE.

### Organization

The rest of this paper is organized as follows. “Related work” section describes the related work in this area. “ System description and overall design” section provides the system overview. “System architecture design” section describes the architectural design. “Implementation” section describes the implementation of the EHR system. "Discussion" section concludes by describing the future scope of ABE and the overall conclusions of this research effort.

## Related work

### Electronic health record system

An EHR system is a digital platform used to store and manage patient data electronically. It includes patients’ medical backgrounds, diagnoses, prescriptions, therapies, test outcomes, etc. EHR systems substituted the role of paper-based records and gave users access to a centralized database for patient data access and exchange. They facilitate effective drug administration, enhance communication and collaboration in healthcare settings, and provide clinical decision-support tools to assist decision-making. EHR systems are essential for promoting patient safety, increasing efficiency, and supporting evidence-based medicine while allowing seamless care coordination and community health management objectives.

Automating medical health record management systems has been the focus of past research^18–21^. Cloud-based EHR systems have been adopted for efficient health data management and control^22,23^. The flexibility, high availability, and low cost of cloud services explain this. The privacy and security of medical data, being the crucial factor, have seen various approaches being proposed^19,20,24^. ABE suggested by Narayan et al.^21^ has been a popular choice in healthcare systems to protect the privacy of EHR data from external threats and the Cloud Service Provider (CSP). Joshi et al. presented cloud-based EHR systems^25,26^ that used CP-ABE to encrypt patient records and ABAC for access control. Walid et al. used CP-ABE in their EHR systems^27–30^ to encrypt patient records. Qin et al. in^31^ and Liu et al. in^14^ used KP-ABE in their EHR systems to encrypt patient records. Warren et al. in^32^, Tembhare et al. in^33^, Mhatre et al. in^34^, and Dixit et al. in^35^ used MA-ABE to encrypt patient records. Likewise, many EHR systems in current literature use either CP-ABE, KP-ABE, or MA-ABE encryption scheme in their system^36–41^.

**Figure 2:**
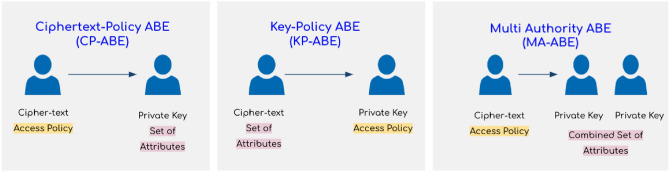
Fundamental distinction between CP-ABE, KP-ABE, and MA-ABE scheme.

### Attribute-based encryption

ABE^42^, introduced by Sahai and Waters, is a cryptographic approach that offers fine-grained access control over encrypted data. It is regarded as one of the renowned security standards for EHR systems^21,43,44^. It enables data owners to specify the attributes necessary for access while encrypting their data. The data can be decrypted and accessed only by authorized users with the required attributes. ABE provides adjustable access rules that enable access control based on different combinations of attributes, such as user, time, or location-based attributes. It eliminates weaknesses and guarantees data security. It uses a certain set of attributes to create the private key and a different set of attributes to encrypt data. The ciphertext can only be deciphered if the two sets of attributes match, according to the threshold setting. ABE has been divided into CP-ABE^5^, and KP-ABE^6^ due to a lack of expressibility. CP-ABE associates access policies with ciphertexts, allowing data owners to specify attribute-based policies written in terms of user attributes as a boolean expression for decryption. Several CP-ABE schemes are proposed in current literature^45–49^. In Contrast, KP-ABE associates access policies with users’ secret keys, simplifying the encryption process and granting users access to data based on their predefined key policies. There are several KP-ABE schemes proposed in current literature^50–53^. Comparatively, CP-ABE offers more flexibility in access control, while KP-ABE is often preferred for scenarios where users have predefined access policies. Depending on the particular needs and required degree of flexibility in the access control system, the choise of scheme varies.

In occasions when there are many authorities, MA-ABE^7^, an extension of ABE, enables decentralized access control. Each authority in MA-ABE is responsible for managing its own set of attributes. Access policies are defined using a mix of attributes from several authorities. This allows for collaboration and data exchange between several organizations or groups while keeping fine-grained control over access to encrypted data. Several MA-ABE schemes are proposed in current literature^54–57^. By providing a distributed architecture, MA-ABE increases the flexibility and scalability of ABE, making it suited for applications that call for the safe and regulated sharing of sensitive information across various authorities. MA-ABE can use either CP-ABE or KP-ABE as its underlying encryption scheme. Figure 2 shows the key distinctions between the three encryption schemes.

### Access control

Access control is the process of identifying a person and deciding their security access to electronic systems based on the organization’s rules and regulations. A sequence of actions is followed to ensure a user can access the requested resources. The typical action sequence is Identification, Authentication, and Authorization. There are different access control models, such as Mandatory Access Control (MAC)^58^, Role-Based Access Control (RBAC)^59^, Discretionary Access Control (DAC)^60^, and attribute-based access control (ABAC)^61^. MAC is a type of access control in which the operating system restricts a subject’s or initiator’s ability to access or conduct a general action on an item or target. MAC is related to two security models: Biba^62^ and Bell-LaPadula^63^. Biba is a model in which a user with low clearance can read higher-level information, and a user with high clearance may write for lower clearance levels. In contrast, Bell-LaPadula is a model in which a user at a higher level can only write at that level but can read at lower levels. RBAC is a policy-agnostic access-control technique based on roles and privileges. Role permissions, user roles, and role-role linkages are just a few RBAC components that make user assignments straightforward. Users are assigned roles dynamically via Rule-Depending Access Control based on criteria established by the custodian or system administrator. Several EHR systems use RBAC model in their systems^64–66^. DAC provides individual control over any items they possess and the programs connected with them. As a result, DAC has two significant flaws. First, it gives the end-user total control over the security level settings for other users, resulting in people having more access than they should. Second, the end-permissions users are passed down to the various programs they run. This implies the end-user may unknowingly execute malware, and the virus could use the user’s high-level privileges. ABAC, also known as policy-based access control for Identity Access Management, is an access control paradigm in which users’ access permissions are provided based on policies that combine attributes. The ABAC model incorporates the advantages of DAC, MAC, and RBAC while also expanding on their constraints. The concept is built around general properties that are used to store DAC identities and access control lists, MAC clearances and classifications, and RBAC roles. Because any number of attributes may be added inside the same extensible framework, the paradigm provides additional flexibility in policy definitions. It also addresses the inadequacies of the fundamental RBAC paradigm. Several EHR systems use ABAC model in their systems^25–30,35^.

### Semantic web technology

Semantic web technology refers to standards, tools, and approaches that improve the web with structured, machine-readable information. The knowledge graph, which serves as the reasoning component in our system, was created using semantic web technologies. Semantic web technology allows data to be tagged with machine-understandable meta-data, automating their retrieval and utilization in the appropriate settings. It comprises tools for reasoning about these descriptions and languages, such as Resource Description Framework (RDF)^67^ and Web Ontology Language (OWL)^68^ for constructing ontologies and expressing meta-data using these ontologies. There are numerous ways to express OWL semantic web knowledge in rule formats, such as N3-logic rules^69^ and SWRL rules^70^. These technologies may be leveraged to establish standard semantics for service information and policies, allowing any agents who comprehend the fundamental semantic web technologies to communicate efficiently and exchange services with each other.

A few essential design requirements exist for systems built using semantic web technologies. The primary demand is for a representation to facilitate interoperability at both the syntactic and semantic levels. OWL’s well-defined semantics, based on first-order logic and model theory, provide confidence that their results will always be accurate. OWL has a significant advantage over many other knowledge-representation systems in that it has well-defined subset profiles that ensure sound and complete reasoning at different levels of reasoning complexity and is made to work with common implementation technologies, such as OWL QL for databases and OWL RL for rule-based systems. The need for a language with good web integration is another design requirement. OWL is constructed using fundamental web standards and protocols and is constantly changing to be compatible with them. HTML pages can contain RDF and OWL knowledge that several search engines, including Google, can find and process. RDF is also compatible with Microdata, an HTML specification developed by the Web Hypertext Application Technology Working Group and used to nest semantic assertions within preexisting web page content.

### Regulatory policy

Many healthcare regulations are established and enforced by various entities at the federal and state levels in the United States. HIPAA^71^ is the primary act that governs the protection of patient data. The main goal of HIPAA is to protect the privacy of medical information that can be used to identify a specific individual. While the HITECH Act^72^ allows the sharing of patient data for medical services, it also requires the HIPAA Act to be enforced. Notably, the acts do not specifically list the encryption methods to be used; instead, they list encryption as an ”addressable” rather than a necessary requirement. When it comes to sharing electronic safe health information (ePHI), this categorization has given rise to conflicting interpretations and has become contentious.

According to the HL7 EHR functional model^16^, EHR systems must abide by the regulations or guidelines set out to restrict access to and safeguard the privacy and security of EHR data. Security procedures protect against the loss, alteration, and destruction of data. The primary security functions are user authentication, authorization, access control, patient access management, non-repudiation, secure data exchange, safe data routing, information attestation, patient privacy and confidentiality, and information attestation.

## System description and overall design

**Figure 3:**
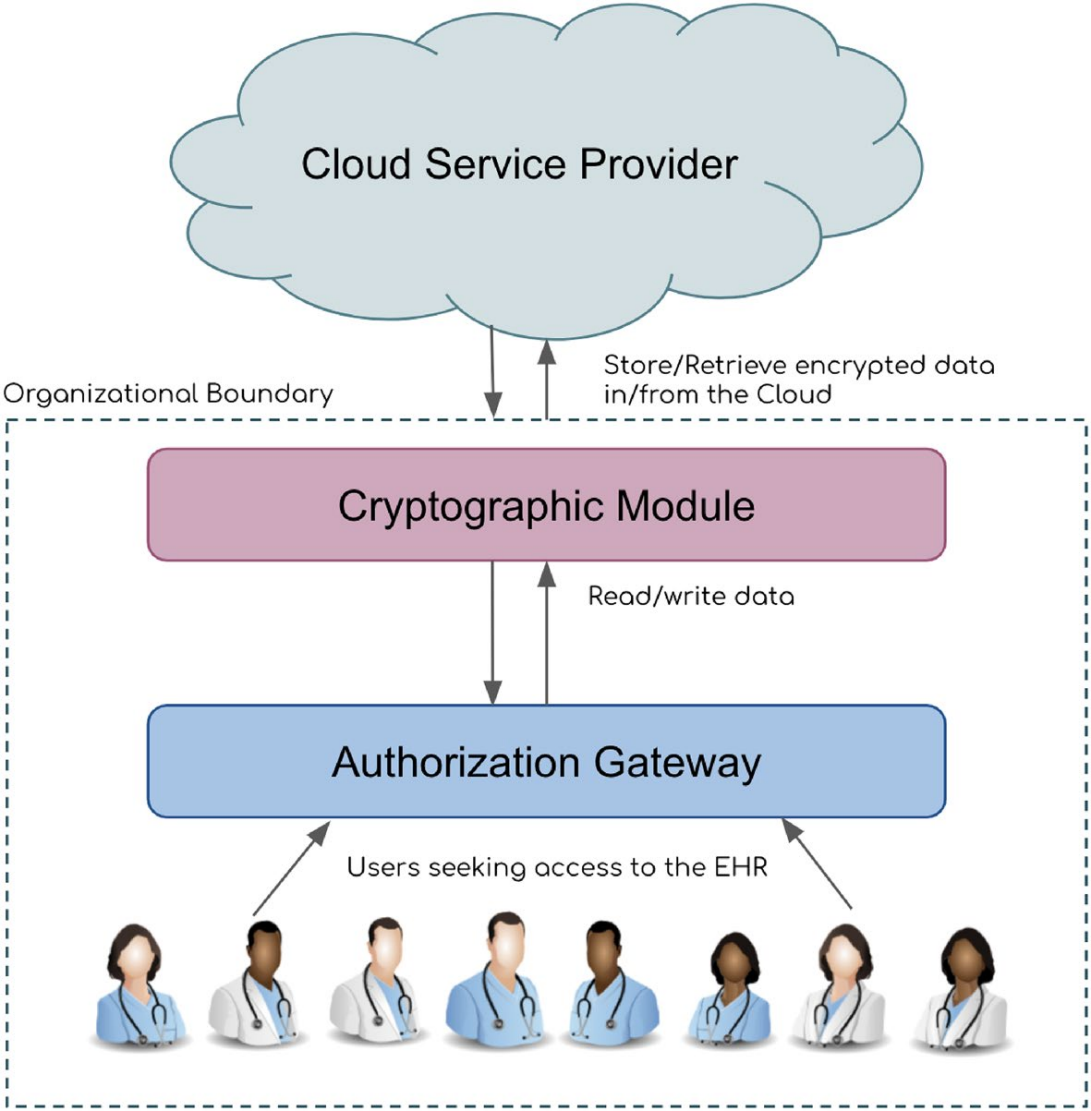
Multiple layers of the EHR systems.

We developed cloud-based EHR systems that are highly secure and provide data access flexibility to end users. Using semantic web technologies, like OWL, we built three separate EHR systems for the different ABE techniques: CP-ABE, KP-ABE, and MA-ABE. We designed three different knowledge graphs for the systems that store the patient information, encrypted medical data, user and their attributes, and other properties in the systems. We referenced the HL7 function model in our design. We collaborated with our colleague, Dr. Michael A. Grasso, an Assistant Professor at the University of Maryland School of Medicine, to understand how EHR systems are used in hospitals. His insight helped us in designing the process flow of our system.

We started by focusing on implementing a policy-defined ABAC model for the EHR systems and designed a user-id/password authentication scheme. Our systems comprise multiple stakeholders, including doctors with various specializations, nurses, patients, emergency service personnel, and pharmacists. Our systems do not support data exchange and routing, which is part of our future work. Our systems are designed based on the principles of edge computing^73^, so all computations on data are performed within the organizational perimeter. The overview of our systems is shown in Fig. 3. Our systems have four levels. In level 1, users request access to the EHR system. Users are authenticated in level 2, and proposed actions are evaluated with respect to access rules, policies, and user attributes. Any updates to the data are made at level 3, and these updates are re-encrypted based on the underlying ABE scheme. At level 4 is the CSP, where the data is sent and stored. Levels 1 to 3 lie within the organizational border and level 4 is outside. The entities outside the border are considered untrusted according to the edge computing principle. Each user passes through a thorough screening process through the Authorization Gateway, which uses ABAC to control access to the data. On passing the access control check with the Authorization Gateway, the user request is sent to the Cryptographic Module, where data is decoded with the help of secret keys. If the data is modified, it is re-encrypted and inserted into the nodes of the knowledge graph. The CSP acts like data storage and stores the knowledge graph, which details the properties of each entity and the relationship between them in the medical organization ecosystem.

## System architecture design

**Figure 4:**
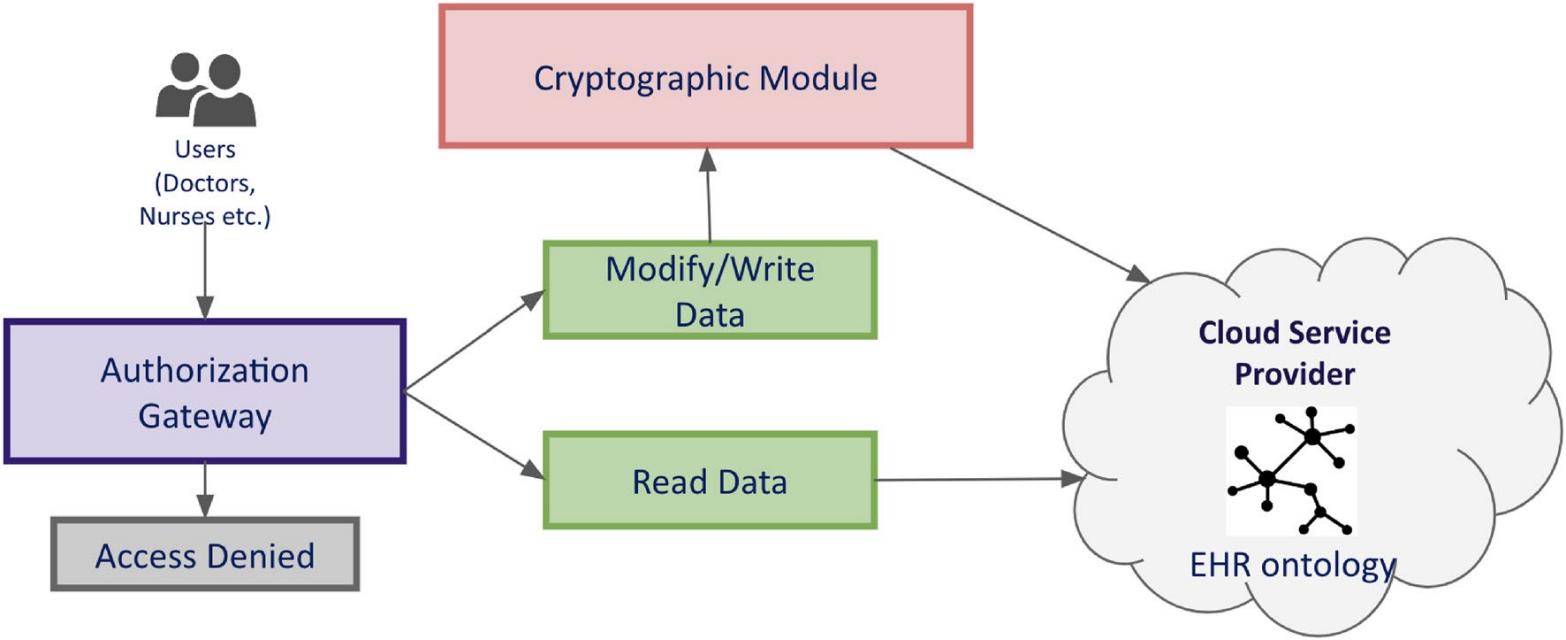
System Architecture.

The system architecture shown in Fig. 4 consists of three main modules: Authorization Gateway, Cryptographic Module, and EHR knowledge graph. It is the same for the three EHR systems, with the only difference being the encryption scheme used in the Cryptographic Module. The data flow in the systems is as follows. Users first log in to the system with their own credentials. The system performs a comprehensive check to authenticate the users using the Authorization Gateway. Once the check is passed, the Gateway determines the user’s access types: read, write, or modify based on the organization policy described as a boolean expression in terms of user attributes. The user then chooses to read or write an EHR. Once the user action is completed, the data is encrypted with the help of the Cryptographic Module. The Cryptographic Module uses ABE to encrypt patient data. It extracts the user attributes by querying the knowledge graph stored in the cloud. It completes the encoding operation using the user attributes and the secret keys. The encrypted text is inserted into the EHR knowledge graph stored within the CSP by creating a new node.

Following is the mathematical representation of the system implementation using CP-ABE.

User set $$U = \{U1, U2, \ldots , Un\}$$

User Attribute Set $US = \{UA1, UA2, UA3, \ldots , UAn\}$

EHR set $$E = \{E1, E2, \ldots , En\}$$

EHR attribute set $ES = \{EA1, EA2, EA3, \ldots , EAn\}$

EHR Fields Set $EF = \{EF1, EF2, \ldots , EFn\}$

EHR Fields Subset $EFS \subset EF$

Policy set $$PS = \{PS1, PS2, \ldots , PSn\}$$

Decryption Policy set $DS = \{DS1, DS2, \ldots , DSn\}$

$\forall$ User *U*, $\exists$ User Attribute Set *US*

For evaluating access decision:

For each User *X* $\wedge$ EHR *Y* $\wedge$ EHR Fields Set *EF*, if *US* satisfies any one policy from $PS \rightarrow$ Read and/or Write (User *X*, EHR *Y*, *EFS*).

For encryption using ABE:

For each User *X* $\wedge$ EHR *Y*, $\exists$ Fields Subset *EFS*, *X* $\wedge$ *Y* $\wedge$ User Attribute Set *US* $\wedge$ $EFS \rightarrow$ Encrypted EHR field where $US \subset DS$.

For decryption using ABE:

If User Attribute Set $US \subset DS$ , *US* $\wedge$ $EF \rightarrow$ Decrypted *EFS*.

Our MA-ABE system also uses CP-ABE to encrypt patient records. The mathematical representation of the MA-ABE is similar to the above, with the only difference of having combined keys from multiple authorities to encrypt and decrypt patient records.

The mathematical representation of KP-ABE follows an analogous structure to CP-ABE. The main difference lies in the association of access policies that can be listed as follows:

- In KP-ABE, access policies are associated with the secret keys of users rather than the ciphertexts themselves.
- The encryption process generates ciphertexts without specific access policies, and the user’s key specifies the policy that the ciphertext should satisfy for decryption.

Other elements, such as user sets, attribute sets, EHR sets, and field sets, remain similar in both CP-ABE and KP-ABE.

We define the different components used to develop the system in the following subsections.

### Authorization gateway

The Authorization Gateway uses a database to authenticate the users and a knowledge graph for access control. It uses ABAC in all three EHR systems, and the policies are set to ensure the right permissions for authenticated users. The Gateway extracts the user attributes along with the EHR fields from the knowledge graph using semantic web technologies. It regulates access down to the field level of the EHR.

Every organization has its own set of rules for data access which incorporates the confidentiality policy of the organization. Moreover, HIPAA and the HITECH Act are common rules and standards for all medical organizations. The policy in our system is defined as a boolean expression in terms of user attributes in the knowledge graph. The knowledge graph provides the attributes for the Authorization Gateway and makes the Crypto Module work. The Authorization Gateway writes dynamic SPARQL^74^ queries to pull the user attributes from the knowledge graph and make access control decisions. Moreover, instead of evaluating the access decision for a complete EHR, the Authorization Gateway evaluates the access decision at the field level of an EHR. Thus, a user may not access the complete EHR but may be granted to specific fields based on the attributes. For implementation and prototyping purposes, we have used the HIPAA Act as the policy that determines access control over patient EHRs.

### Cryptographic module

The Cryptographic Module is the essential component of the system, and it is responsible for any crypto operation, including protecting the data against any leaks and threats. The Module uses ABE to encrypt patient data. We implemented CP-ABE, KP-ABE, and MA-ABE in three systems, respectively. The Module uses user attributes from the EHR knowledge graph to perform the crypto operation at the EHR field level instead of the traditional approach of using ABE at the document level. Moreover, since the MA-ABE EHR system involves multiple authorities, the Module uses the combined attributes and keys from the authorities to perform crypto operations.

The Cryptographic Module has another critical task of producing the secret and public keys needed for the systems. It does it by obtaining the user attributes from the knowledge graph and policies from the Authorization Gateway. Data read/write also happens within the Module. The Module is needed for any system function and works as a co-ordinator between the Authorization gateway and the EHR knowledge graph.

The knowledge graph supports the Cryptographic Module module by delivering the correct user attributes for any system function, like reading or writing patient data. The Cryptographic Module writes dynamic SPARQL queries to obtain the user attributes and EHR fields. The following shows a simple SPARQL query to retrieve the Allergies field data of a patient with id 100 from an encrypted node.
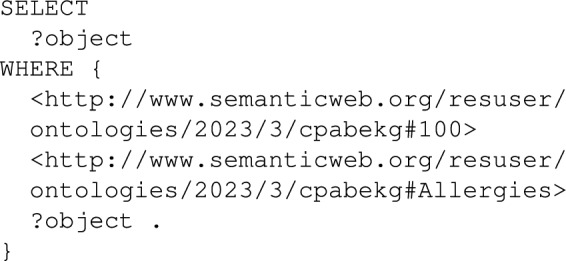


Whenever a patient EHR field is updated, the current node in the knowledge graph is deleted, and then a new node is created where the data is inserted. The following SPARQL queries are shown as simple examples when the Allergies field of the patient with id 100 is updated.
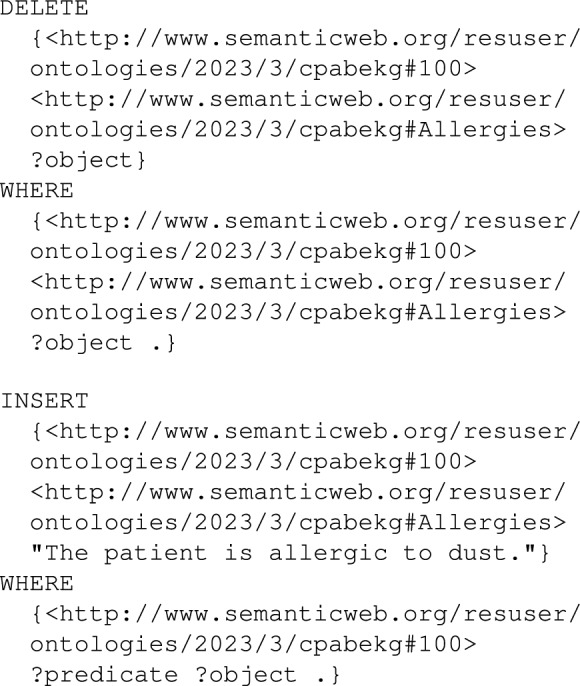


### EHR knowledge graph

**Figure 5:**
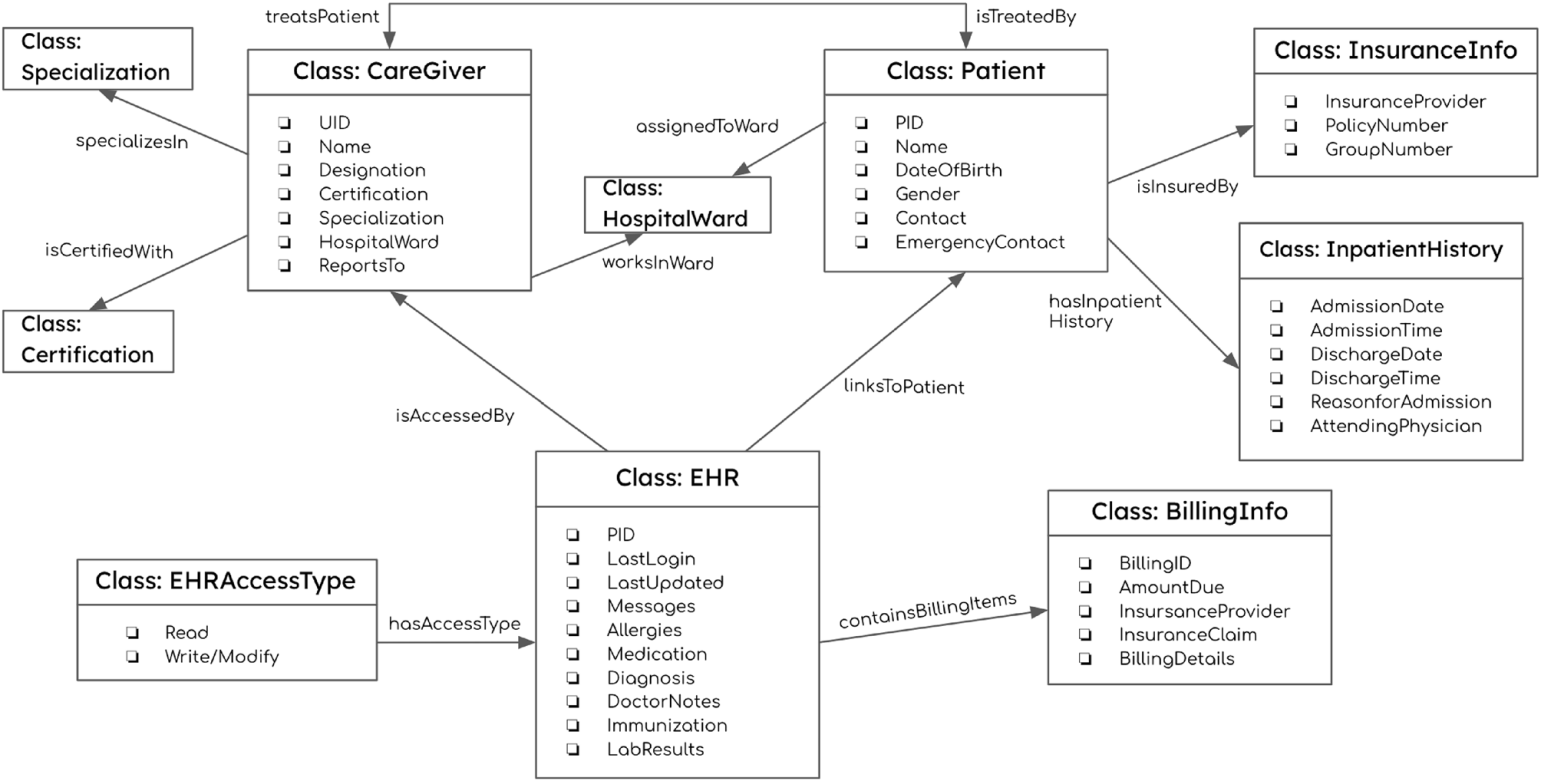
Knowledge Graph used in the EHR systems.

The knowledge graph used in the systems is shown in Fig. 5. It was designed by referencing the HIPAA knowledge graph^75^ and the medical standards specified by the National Institutes of Health, the National Healthcare Association, and HealthIT.gov. The graph records medical organization users like Doctors, Nurses, Patients, and other medical users and encrypted data. It stores Certifications like MD (Doctor of Medicine), PharmD (Doctor of Pharmacy), EMT-B (Emergency Medical Technician-Basic), and RN (Registered Nurse). Hospital Wards like Oncology, Pediatric, and Specializations like Cardiology, Gynaecology, and Ophthalmology are also stored. Likewise, EHR fields like Billing Information, Doctor Notes, Lab Results, Immunization Dates, Diagnoses, Allergies, and Medications are stored in the graph as data properties to store patient information. The EHR field access is controlled by the graph using ABAC model to protect privacy. The Certification, Specialization, and Hospital Wards stored in the graph serve as the attributes of a user.

## Implementation

Our EHR systems are web-based applications developed in Python to manage field-level ABE and access control of patient data. The systems use ABAC to confirm that the right users can access the right data. The systems use ABE to ensure robust data encryption techniques. The secret keys for the CP-ABE and MA-ABE systems are produced using the user attributes, whereas, in the KP-ABE system, the secret keys are tagged with an access policy and produced accordingly. We developed the systems so that each component performs its functions independently and, as a whole, serves as a suite of services. Our design encourages the reuse of sub-modules when creating new systems that call for the same functionality.

The systems were built using open-source tools, Python language, libraries, and APIs. They were developed using the Python Django framework based on the foundations of the Model-View-Controller (MVC) architecture^76^. We created the systems utilizing the views, models, templates, and URLs of the frameworks to give patients and medical users quick, secure, and safe access to the EHRs. Our system also uses semantic web technologies. We have built knowledge graphs using Protege [protege.stanford.edu] and used SPARQL queries to read/write on the knowledge graphs. An open-source Python library named rdflib is the intermediary that helps connects the Python-based systems and the OWL ontologies.

### Field-level ABE encryption

The most vital module of our systems is the Cryptographic Module that uses ABE. Since we have three systems with different ABE techniques, we describe the system crypto functions of each separately in the following sub-sections.

#### CP-ABE

CP-ABE assigns a specific decryption policy on any document, which is a logical expression based on the attributes of the users. The document can be decrypted and used by users whose attributes comply with the decryption policy. The CP-ABE library offers four functions: cpabe-setup, cpabe-keygen, cpabe-enc, and cpabe-dec. The cpabe-setup function produces the public key and a master secret key required for subsequent operations. The cpabe-keygen function generates a private key with a given set of attributes. It is the private key for the user to encrypt/decrypt the document. The cpabe-enc function encrypts a document according to a decryption policy expressed as a logical attribute expression. If the user attributes are satisfied, the cpabe-dec function decrypts an encrypted document using the private key generated by cpabe-keygen.

#### KP-ABE

KP-ABE assigns a specific decryption policy on the user secret key, which is a logical expression based on the attributes. Any document is encrypted with all the attributes in the system and can be decrypted if the two sets of attributes overlap. The KP-ABE library supports four functions: kpabe-setup, kpabe-keygen, kpabe-enc, and kpabe-dec. The kpabe-setup function produces the public key and master secret key required for subsequent operations. The kpabe-keygen function generates a private key which is tagged with an access policy expressed as a logical attribute expression. It is the private key for the user to encrypt/decrypt the document. The kpabe-enc function encrypts a document by using the public key and all the user attributes in the system. If the access policy satisfies, the kpabe-dec function decrypts an encrypted document using the private key generated by kpabe-keygen.

#### MA-ABE

MA-ABE assigns a specific decryption policy on any document, which is a logical expression based on the attributes of the users from different authorities. The document can be decrypted and used by users if attributes of users from different authorities comply with the decryption policy. The MA-ABE library supports the five functions: maabe-setup, maabe-authsetup, maabe-multiple-attributes-keygen, maabe-encrypt, and maabe-decrypt. The maabe-setup function produces the public parameters. For each authority, the maabe-authsetup function produces the public key and secret key by taking the attributes and public parameters. Likewise, for each user from an authority, maabe-multiple-attributes-keygen function produces the user keys by taking public parameters, secret key, global user identifier, and user attributes. The maabe-encrypt function encrypts a document based on the access policy described in terms of user attributes from different authorities. The maabe-decrypt function decrypts a document when combined user keys produced by their attributes satisfy the access policy.

Please refer to the original implementation of CP-ABE^5^, KP-ABE^6^, and MA-ABE^7^ for further details.

### Security models for ABE schemes

Security models define the framework for assessing how well a scheme protects sensitive information. They provide a systematic way to evaluate and test the scheme’s resilience against attacks. We describe the security models of the encryption schemes used in the Cryptographic Module in the following sub-sections.

#### CP-ABE

The security model for CP-ABE is designed to ensure the confidentiality of data while allowing flexible access control based on attributes. The model includes several components: correctness, attribute-hiding security, access policy security, adaptive chosen-ciphertext security, key policy security, and collision resistance. Correctness guarantees that data are encrypted and decrypted by the specifications of the CP-ABE scheme. The encryption and decryption procedures should generate anticipated outcomes without any glitches. Attribute-hiding security guarantees that an adversary cannot discover any information about the attributes linked to a ciphertext or the user’s secret key, even after seeing several ciphertexts. Access policy security guarantees that the data can only be decrypted by users with the attributes defined by the access policy linked with the ciphertext. It should be impossible for adversaries lacking the necessary attributes to get access. Adaptive chosen-ciphertext security addresses how the CP-ABE system withstands adaptive chosen-ciphertext attacks. Key-policy security ensures that the CP-ABE scheme is secure even when the adversary has access to the secret keys for some users, as long as these secret keys do not violate the specified access policies. The collision resistance security model guarantees that even when users collaborate to combine their attributes, the security of the CP-ABE scheme remains intact.

#### KP-ABE

The security model for KP-ABE is formulated to ensure the secure and reliable functioning of the encryption and decryption processes in a key-policy attribute-based encryption scheme. The model includes several components: correctness, attribute-hiding security, access policy security, adaptive chosen-ciphertext security, key policy security, and collision resistance. Correctness ensures that the encryption and decryption functions produce the intended results without errors. Attribute-hiding security guarantees that an adversary, even with knowledge of the ciphertext and the attributes of some users, cannot know additional information about the attributes of other users. Access policy security ensures that only users possessing the appropriate attributes specified in the access policy can decrypt the ciphertext successfully. Adaptive chosen-ciphertext security addresses the resilience of KP-ABE against adaptive chosen-ciphertext attacks. Key policy security ensures that the scheme remains secure even if an adversary has access to some users’ secret keys, provided that these secret keys comply with the key policies. Collusion resistance ensures that even if users with different attributes collaborate, the security of the scheme remains intact.

#### MA-ABE

The security model for MA-ABE is designed to handle the unique needs of scenarios with multiple authorities managing various attribute sets. The security model attempts to give a flexible access control mechanism while guaranteeing the confidentiality and integrity of data. The model includes several components: correctness, attribute-hiding security, access policy security, adaptive chosen-ciphertext security, key policy security, collision resistance, and inter-authority security. Correctness ensures that the encryption and decryption functions in MA-ABE produce the intended results without errors. Attribute-hiding security guarantees that an adversary, even with knowledge of the ciphertext and the attributes from different authorities, cannot know additional information about the attributes of specific users. Access policy security ensures that only users possessing the appropriate attributes specified in the access policy can decrypt the ciphertext successfully. Adversaries lacking the required attributes should not gain unauthorized access. Adaptive chosen-ciphertext security addresses the resilience of MA-ABE against adaptive chosen-ciphertext attacks. Key policy security ensures that the scheme remains secure even if an adversary has access to the secret keys from some authorities, provided that these secret keys comply with the access policies. Collusion resistance ensures that even if users from different authorities collaborate, the security of the scheme remains intact. Inter-authority security ensures that each authority follows the protocol strictly and does not maliciously compromise the security of the overall system.

Please refer to the original implementation of CP-ABE^5^, KP-ABE^6^, and MA-ABE^7^ for the proof of the security models.

### Dataset description

We have used the MIMIC-III^77^ dataset to create our synthetic graph dataset of different sizes for the various encryption schemes. We used data with 20,000, 40,000, 60,000, 80,000, and 100,000 patient instances for our experiments. Each patient has several fields in their EHR based on their medical histories, such as Allergies, Billing Details, Diagnosis, Doctor Notes, Medication, Lab Results, and Immunizations. The patient data is stored as encrypted nodes in the knowledge graphs. By following the edge computing principles, all computations on the data are done within the organization’s perimeter and kept in the CSP. We have 20 medical users, like Doctors, Nurses, etc., in each of the EHR systems. Each medical user has certification, specialization, and hospital ward attributes. Various users with distinct attributes have unique access to the EHR fields.

### Systems evaluation and comparison

We designed a proof of concept prototype to assess the EHR systems. Let’s consider the CP-ABE based system. Assume a doctor named Mickel requests access. The request is assessed in the Authorization Gateway module; the username and password are validated against the database; the EHR ontology offers unique attributes for Dr. Mickel based on the defined ABAC policy. If Dr. Mickel plans to retrieve the EHR of a patient named Andy, the request is completed in the Cryptographic Module using the secret keys and the ABE encryption scheme. If Andy’s data is modified, it is re-encrypted in the Cryptographic Module and inserted as a new node in the knowledge graph. The KP-ABE system works similarly. However, in the MA-ABE system, public keys produced by the different authorities are combined and used to encrypt data. Likewise, user secret keys from various authorities are combined and used to decrypt an encrypted patient record.

**Table 1:** Query Performances of the different ABE schemes listed in seconds.

Number of patient records	CP-ABE			KP-ABE			MA-ABE		
	Encrypt	Decrypt	Delete	Encrypt	Decrypt	Delete	Encrypt	Decrypt	Delete
20,000	0.0343920	0.0449030	0.0029766	0.1594585	0.3364872	0.0026686	0.0618480	0.0847001	0.0033932
40,000	0.0363633	0.0462562	0.0036356	0.1504242	0.3387392	0.0032237	0.0614241	0.0822896	0.0033574
60,000	0.0347301	0.0444610	0.0030626	0.1727807	0.3111324	0.0027198	0.0622311	0.0833372	0.0028348
80,000	0.0357982	0.0447646	0.0032266	0.1375874	0.3081261	0.0026554	0.0620117	0.0828410	0.0034529
100,000	0.0344876	0.0462038	0.0030785	0.1537148	0.3186670	0.0025938	0.0596107	0.0851838	0.0034008

**Table 2:** Number of Keys in CP-ABE, KP-ABE, and MA-ABE schemes.

Key type	CP-ABE	KP-ABE	MA-ABE
Public key	1	1	2
Master key	1	1	N/A
Secret key	1	1	2
Public parameter	N/A	N/A	1
User key	N/A	N/A	2

**Table 3:** Size of each key used in the ABE schemes.

Key type	CP-ABE	KP-ABE	MA-ABE
Public key	1 KB	1 KB	658 Bytes
Secret key	5 KB	1 KB	270 Bytes

We evaluated the performance of encrypting, decrypting, and deleting an EHR field in the three EHR systems. For the MA-ABE system, we implemented CP-ABE as an underlying ABE scheme and considered two authorities. Table 1 shows the performance of the queries for different data sizes. The query performances are listed in seconds using an average of ten queries. The encrypt query in the table means encrypting an EHR field, creating a new node in the knowledge graph, and inserting the encrypted data into the graph. The decrypt query in the table means decrypting an EHR field containing encrypted data. The delete query means to delete an encrypted node in the knowledge graph. We can see in the table that the query performance in each column is almost the same, meaning data size does affect the performances, and this proves that graph-based systems are highly scalable. We also observe from the table that CP-ABE has the best encrypt and decrypt performance compared to KP-ABE and MA-ABE. Moreover, we can also see that MA-ABE encrypt and decrypt performance is almost double that of CP-ABE. This is obvious as the MA-ABE system was developed using CP-ABE as an underlying scheme, and since we have two authorities that took part in encryption/decryption, the performance is almost doubled. The delete performance is almost the same for CP-ABE and KP-ABE and slightly higher for MA-ABE.

We have listed the number of different keys produced and used by each ABE system in Table 2. We can see that CP-ABE and KP-ABE each produced one public key, whereas MA-ABE generated two public keys because of having two authorities. The MA-ABE system did not produce a master key, but CP-ABE and KP-ABE each produced one master key. The MA-ABE system produced two secret keys due to having two authorities, and CP-ABE and KP-ABE systems each produced one secret key. The public parameter is only present in the MA-ABE system. Likewise, user keys are only present in the MA-ABE system. However, the user private keys in the CP-ABE and KP-ABE systems are known as the secret key, which is used to encrypt data. At the same time, the MA-ABE system has both user keys and secret keys. The system uses the secret keys to create the user keys and user keys are then used to encrypt data.

The size of a public and secret key common in all systems is listed in Table 3. The size of each public key in the CP-ABE and KP-ABE systems is the same and higher than that of the MA-ABE system. The size of each secret key in the CP-ABE system is almost five times that of the KP-ABE system and nearly nineteen times that of the MA-ABE system.

## Discussion

ABE has been widely used in EHR systems, and selecting the appropriate ABE scheme involves careful consideration of the unique demands of the environment. Our experiments show that CP-ABE constantly demonstrates low encryption times, making it a good option when encryption speed is critical. Though slower than CP-ABE, KP-ABE exhibits steady decryption speeds and moderate encryption. MA-ABE, on the other hand, has stable decryption times that are similar to CP-ABE and moderate encryption times with occasional fluctuation.

The number of keys in CP-ABE and KP-ABE is the same; each has one public key, one master key, and one secret key. By adding a second user key and public parameter, MA-ABE, on the other hand, presents a more sophisticated structure that might affect the distribution of keys and system complexity. However, With a smaller public key (658 Bytes) and a much smaller secret key (270 Bytes), MA-ABE is recognized for having compact key sizes, which can improve transmission and storage efficiency.

The optimal applications of the ABE schemes are diverse and require a thorough assessment of the particular requirements in the system. CP-ABE is ideal when data access control policies are attribute-centric, allowing for flexible and fine-grained access control over who can access what based on specific attributes. This proves advantageous in scenarios where sharing sensitive information with professionals possessing specific attributes is crucial. On the other hand, KP-ABE is preferable when efficiency is critical, and access control policies are user-focused, simplifying key management and emphasizing the roles and capabilities of individuals within the healthcare organization. MA-ABE is a valuable option for distributed healthcare ecosystems involving multiple authorities or departments. MA-ABE offers enhanced scalability and flexibility by permitting multiple independent authorities to manage their attribute and collaborate on access control policies. This is especially advantageous when healthcare data spans various entities or organizations. Nevertheless, the benefits of MA-ABE are accompanied by some difficulties, such as the potential for policy conflicts and the complexity of collaboration with multiple authorities.

## Conclusion

This paper describes EHR systems using different ABE techniques such as CP-ABE, KP-ABE, and MA-ABE. It discusses the usability of the schemes within the EHR domain and shows their query performances and some statistics about the keys needed for each technique. All the EHR systems use separate HIPAA-compliant knowledge graphs that help support ABAC and ABE. The graph stores all entities, their attributes, and the relationship between the entities within the medical organization. The patient data is stored as encrypted nodes in the graph, making it highly scalable. Assuming the edge computing principles, the computations on the data are performed within the organizational boundary and before sending the data to the cloud to defend against privacy concerns. The paper can help researchers or organizations using ABE in their systems comprehensively understand the benefits and issues attached to each.

We want to extend our research in several possible directions in the future. We plan to use other publicly available datasets to evaluate and compare the query performances of the systems with the MIMIC-III dataset. We plan to get feedback from physicians by allowing them to use our systems and address any potential shortcomings. We plan to develop an enhanced user interface and run a user-centric evaluation to address usability challenges.

## Data Availability

The codes used for the study are available here: https://github.com/redwanwalid/ABEComparison. The datasets produced and analyzed in the current study are not publicly available on GitHub due to size restrictions but are available from the corresponding author upon reasonable request.
